# Policy brief: Improving national vaccination decision-making through data

**DOI:** 10.3389/fpubh.2024.1407841

**Published:** 2024-12-17

**Authors:** Sandra Evans, Joe Schmitt, Dipak Kalra, Tomislav Sokol, Daphne Holt

**Affiliations:** ^1^Sandra Evans Health Policy, Liverpool, United Kingdom; ^2^Global Health Press, Singapore, Singapore; ^3^The European Institute for Innovation through Health Data, Ghent, Belgium; ^4^European Parliament, Brussels, Belgium; ^5^Coalition for Life Course Immunisation, Brussels, Belgium

**Keywords:** National Immunisation Technical Advisory Groups, National Immunisation Programs, life course immunisation, vaccine policy, vaccine-preventable diseases, big data analysis, AI technologies

## Abstract

Life course immunisation looks at the broad value of vaccination across multiple generations, calling for more data power, collaboration, and multi-disciplinary work. Rapid strides in artificial intelligence, such as machine learning and natural language processing, can enhance data analysis, conceptual modelling, and real-time surveillance. The GRADE process is a valuable tool in informing public health decisions. It must be enhanced by real-world data which can span and capture immediate needs in diverse populations and vaccination administration scenarios. Analysis of data from multiple study designs is required to understand the nuances of health behaviors and interventions, address gaps, and mitigate the risk of bias or confounding presented by any single data collection methodology. Secure and responsible health data sharing across European countries can contribute to a deeper understanding of vaccines.

## Introduction

In the current climate, infectious disease prevention faces significant challenges that are multifaceted and increasingly global in scope:

- Climate change causes changes in weather patterns, expanding the geographical reach of vector-borne infectious diseases like malaria and dengue (1).
- Increasingly complex geopolitical tensions disperse vulnerable populations and disrupt local and global vaccination provision.
- An ageing population and low vaccine uptake mean more people are at risk of experiencing illness from vaccine-preventable diseases (VPDs) (2).
- Inequality, insufficient financing and public sentiment are some factors that disrupt access and hinder effective coverage targets.

Amidst these evolving global health challenges, this review critically assesses the landscape of national vaccination decision-making. It focuses on integrating robust data analysis to inform effective strategies and explores how data-driven approaches can significantly enhance policy recommendations and public health outcomes.

**Figure 1:**
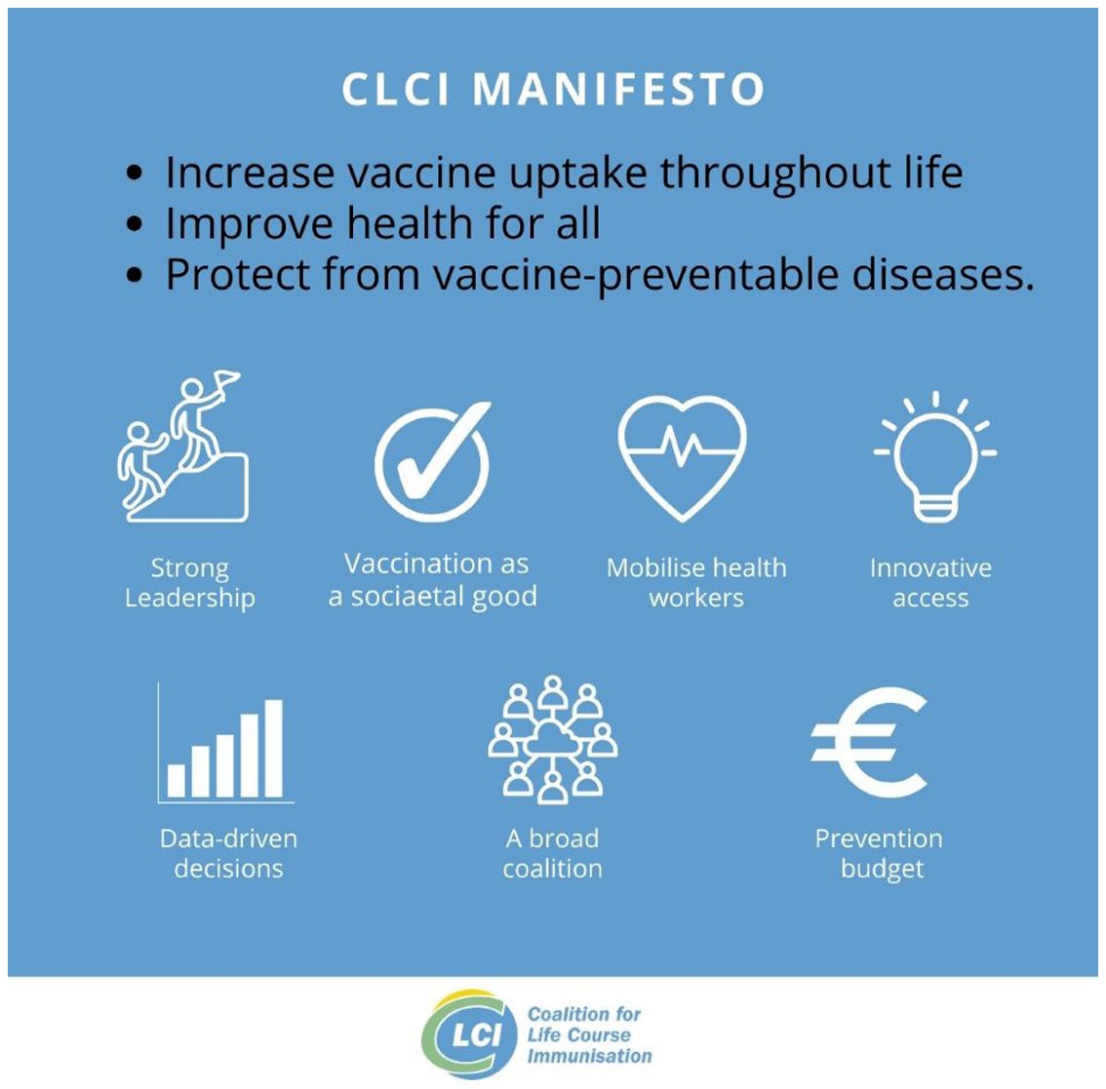
CLCI’s manifesto.

The Coalition for Life Course Immunisation (CLCI) – www.cl-ci.org – is a charity registered in Belgium and the United Kingdom that aims to Increase vaccine uptake in all ages to improve health and protect Europe from vaccine-preventable diseases. CLCI promotes the interpretation of broad data sets to advocate for life-course immunisation strategies. These strategies aim to capture the total value of vaccination across generations, address future health risks and threats, prevent vertical transmission from parent to child, and mitigate long-term health consequences. As shown in Figure 1, the CLCI’s manifesto emphasises adopting data-driven policies and a coordinated approach as essential for advancing life course immunisation. The CLCI recognises the importance of utilising extensive data to uncover valuable insights and identify strategic opportunities for preventive measures, including vaccination. Advances in artificial intelligence, such as machine learning and natural language processing, significantly enhance our ability to use data for shaping policies, tracking diseases, and developing vaccines (3–5).

This review, adopting the perspective of the CLCI, aims to underscore how leveraging data-driven insights can support vaccination policies and improve public health outcomes for all.

In Europe, establishing coordinated life course vaccination schedules aligns with the Treaty on the Functioning of the European Union. Vaccines safeguard the health of all EU citizens, allowing them to safely and freely move and reside across the EU (article 45), and play a critical role in ensuring a high level of human health protection, which should be in all EU policies and activities (article 168) (6).

Ensuring equitable access to vaccination in Europe for all citizens was emphasised in the December 2018 EU Council recommendation on strengthened cooperation against VPDs (7) and the December 2022 EU Council conclusion on vaccination (8).

## The case for expanding sources of data and evidence to inform vaccine policy

National Immunisation Technical Advisory Groups (NITAGs) make vaccination recommendations to the government, who then decide whether to implement them in the national immunisation programs (NIPs). NITAG vaccination recommendations only become available after a review of current scientific medical data (e.g., the burden of disease), sometimes including financial aspects (healthcare budget) by multiple stakeholders. Other factors, such as cultural or religious beliefs and expected public acceptance, are considered, too (9, 10).

As per World Health Organization guidance, almost all EU countries have standardised, clear-cut pathways for vaccine licensure and market authorisation. While most countries have a NITAG, which follows WHO guidance, group composition and practice vary significantly between countries (9, 11).

### The GRADE methodology of assessing evidence quality

**Figure 2:**
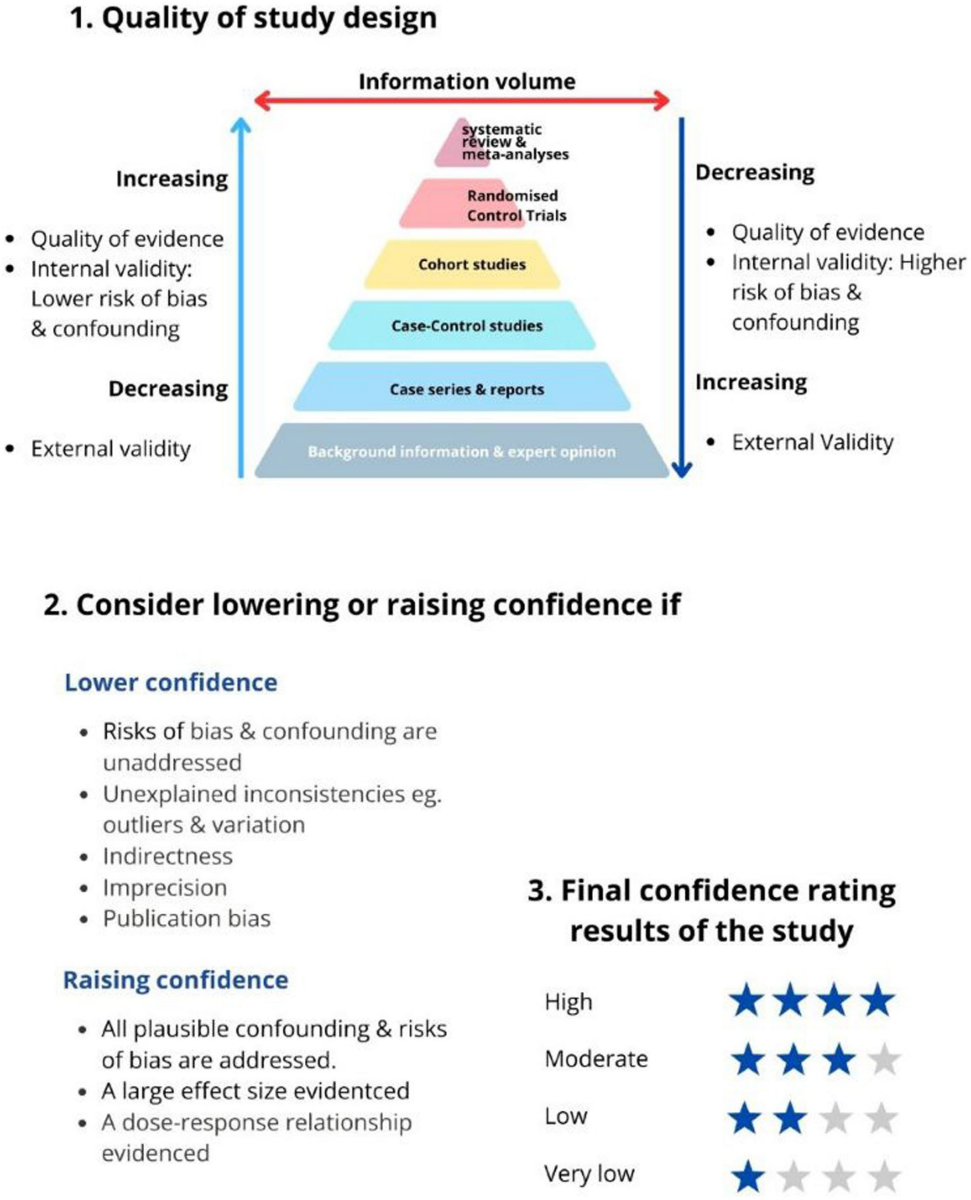
GRADE’s approach to rating the quality of evidence (12).

Most NITAGS use the Grading of Recommendations, Assessment, Development and Evaluation (GRADE) method to evaluate the quality of evidence and make recommendations (12). Figure 2 illustrates the GRADE approach to rating the quality of evidence.

The quality of evidence is based on the research methodology’s ability to remove or control for confounding and bias. For example, data from randomised controlled trials (RCTs) are of high quality, and observational studies are of low quality according to the GRADE ranking (12, 13).

In this approach, RCTs are the golden standard for evidence quality. However, RCT findings are often less generalisable to the real world due to the study’s strict inclusion/exclusion criteria (lack of external study validity) (13). RCTs can also be misinterpreted; for example, if event-driven RCTs are analysed as if they were evaluating incidence rates, it could result in overestimating the vaccine’s effectiveness (14).

### Value of real-world data

Expert opinion is considered low-quality evidence, yet most emerging infectious diseases are discovered because clinicians notice abnormalities (15). The timeliness of decision-making can be hindered by waiting for sufficiently strong GRADE evidence.

Real-world data offers an essential complement to RCT data, spanning more diverse population profiles and vaccination administration scenarios. However, large-scale data is needed to compensate for its diversity and heterogeneous quality statistically.

### Communication of contextual factors

**Figure 3:**
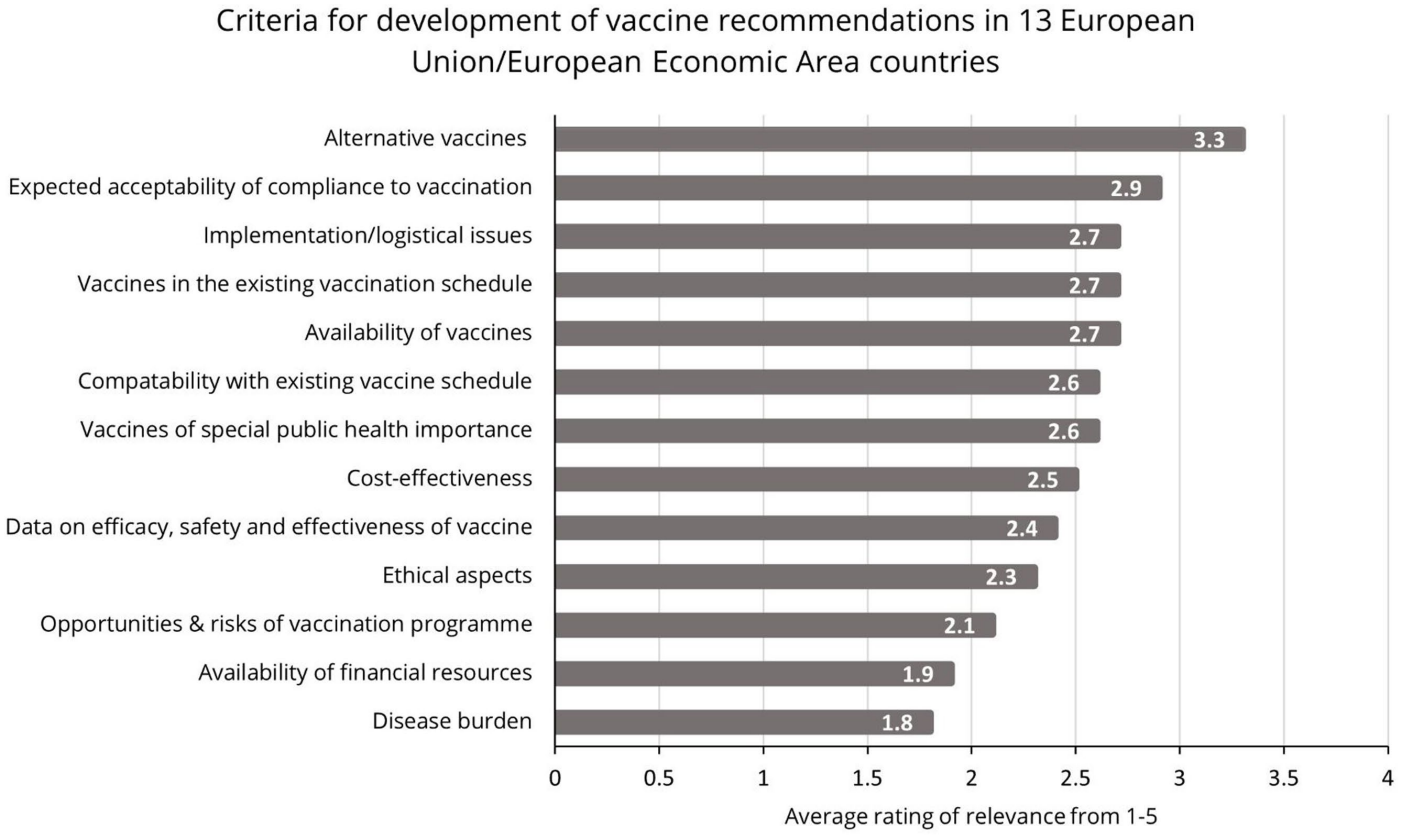
Criteria for the development of vaccine recommendations in Europe (23).

Contextual factors influencing NITAG recommendations, as depicted in Figure 3, are often poorly communicated to the public, who may not understand why one country recommends a vaccine when another does not. Consistent and thoughtful collaboration across stakeholders supports more transparent communications to the public, which can build understanding and trust.

## Policy options and implications

### A life course perspective

Life course immunisation looks at the broad value of vaccination for individuals, communities, and society across multiple generations. This wider lens requires more data power, collaboration, and multi-disciplinary work at various levels.

With this broader perspective, NITAG recommendations should be designed to achieve clear public health outcomes for all and implemented by governments with clear responsibilities and accountabilities. They should include regular evaluation and adjustments as appropriate based on factors, including vaccine uptake and emerging disease burden.

### AI-driven “big-data” analysis in decision-making

Population health is an adaptive, dynamic, and unpredictable system with multiple interdependencies and various factors influencing outcomes (16). Analysing data from numerous study designs, RCTs, real-world data, and conceptual models, is required to understand the nuances of health behaviors and interventions and to mitigate the risk of bias or confounding presented by any single data collection methodology. ‘Big data’ analysis involves modern technologies which interpret large volumes of variable data and spot patterns, often in real time (3). This can facilitate effective rapid response and inform long-term planning, as seen during the pandemic when AI was integral to forecasting COVID-19 spread, contact tracing, pharmacovigilance, and fast testing and detection (4).

The applications of AI are vast in public health research and planning. For example, machine learning approaches such as “neural networks” can improve predictive modelling of complex, nonlinear relationships in data. This can support more accurate forecasting of future trends and predicting disease outbreaks based on historical data (17). Natural language processing has been used to analyse vaccine sentiment via social media (18).

Governments and institutions must look at upskilling NITAGs to effectively interpret insights from large volumes of multi-dimensional data, predictive analytics, and conceptual modelling to forecast vaccination needs and outcomes.

### Availability of harmonised data sets

Combining multiple data sources presents challenges of standardisation and system interoperability. The European Commission launched the European Health Data Space (EHDS) in May 2022, which will be crucial in harmonising data from across Europe, ensuring data quality, compatibility and security. It is a vital pillar of a strong European Health Union and is the first specific data space to emerge from the European data strategy (19).

Gathering and utilising health data depends on overcoming technical, legal, and implementation challenges to ensure the effective transfer of AI models across different healthcare systems. Data privacy and security are significant hurdles to overcome in the context of public trust and vaccine acceptance, calling for a delicate balance between data access and privacy protection.

EHDS will provide a solid legal framework for using health data for research, innovation, public health, policy-making and regulatory purposes. Under strict conditions, researchers, innovators, public institutions, and industries will have access to high-quality health data crucial to developing vaccines. The availability of large-scale health data can support the generation of robust evidence on vaccine effectiveness and safety. Researchers can analyse data across different populations, age groups, and geographical regions to assess the real-world impact of vaccines, identify potential subgroups that may benefit most from vaccination, and detect rare adverse events. Also, EHDS will facilitate information exchange between Member States on vaccination plans and verification of vaccination certificates.

### Multi-stakeholder collaborations

Collaboration and technology can support access to timely and accurate data during the early phase of an outbreak when the chance for containment is highest (20). Global.health is an open-source platform working towards this by facilitating access to real-time, anonymised health data on infectious disease outbreaks. The platform has a 100-day Mission: to provide decision-makers, researchers, and the public with timely and accurate data during the early phase of an outbreak when the chance for containment is highest. With over 100 million verified case records from 130+ countries, it is a comprehensive repository of COVID-19 line-list data. Facilitating the secure and responsible sharing of health data across European countries can contribute to a deeper understanding of vaccine effectiveness, safety profiles, and real-world outcomes.

Bi-directional communication and collaboration on critical data are required for development, and monitoring and evaluation should be enhanced between governments, NITAGs, and Ministries of Health. There are foundations to build on; for example, the WHO sets research and development targets for funders and developers through target product profiles (TPP), which outline the desired ‘profile’ or characteristics of a target product aimed at a particular disease. TPPs state intended use, target populations and other desired attributes of products, including safety and efficacy-related characteristics (21). Such structures and frameworks with strict data ownership and security protocols support a more coordinated approach to improving vaccine impact through broader coverage and strategic use of certain vaccines.

### Communicate nuances in decision-making to the public

The risk of communicating inaccurately is significant. When COVID emerged, reporting journalists unintendedly propagated misunderstanding, which fuelled distrust. For example, the media reported daily disease incidences. However, few countries calculated and communicated scientifically valid incidences with a denominator (persons-tested) that reflected the variation in people getting tested daily based on the ever-changing testing recommendations. Media coverage also focused on the COVID-19 vaccine reducing transmission, which to date is almost impossible for respiratory virus vaccines. These can only “control” respiratory tract infection, i.e., minimise morbidity and mortality (22). Understanding and educating the public and working with key stakeholders, including community leaders, to share trusted, accurate information can inform and empower the public.

Governments might look to their NITAGs, with their expertise and multi-disciplinary composition, to help bridge gaps between various stakeholders, promote transparency, and encourage open dialogue.

## Actionable recommendations

At the national level, NITAGs and governments can work more strategically together and utilise modern tools and resources to build NIPs that span the life course and promote public trust.

- NITAG recommendations for NIPs should be driven by broader public health improvement goals and implemented with clear responsibilities and accountabilities.
- NIPs should include clear communications and regular evaluations of vaccine sentiment, uptake and emerging disease burden.
- A dialogue between multi-disciplinary stakeholders, including healthcare professionals and physicians, should complement the GRADE process to comprehensively address current and future threats alongside opportunities for health promotions of all ages.
- Invest in and upskill NITAGs to utilise data platforms and modern technologies to use large volumes of multi-dimensional data, predictive analytics and conceptual modelling to forecast vaccination needs and outcomes.
- Utilise the multidisciplinary nature of the NITAGs to develop communication channels with different stakeholders, including community leaders, to share data, knowledge, and context regarding vaccine recommendation and impact.

Although health is not a mandate of the European Union, EU institutions can support and guide member states via

- A toolkit or training resource on using AI and modern technologies in data collection and interpretation for policy development.
- Expanding data standardisation protocols that align with the European Health Data Space to ensure data compatibility and ease of analysis.
- Developing transparent and accountable knowledge-sharing channels between member states and private stakeholders to inform future-proofed prevention strategies.
- Support member states with EU-wide dialogue on public sentiment, communication, and raising awareness, including community leaders and reporters.

## Conclusion

A future where everyone, regardless of age or life stage, can be protected from VPDs through comprehensive vaccination programs is underpinned by data-driven decisions. This must involve standardising data sets through platforms like the EHDS, enhancing surveillance systems with AI, and transparent communication between governments, NITAGs, industry, and the public. Future-proofed decision-making requires the upskilling of NITAGs to utilise modern technologies that analyse large volumes of data and generate reliable modelling data to develop recommendations. We must counteract information overload, confusion and misinformation with multidisciplinary stakeholder collaboration, transparency, open dialogue and clear accountability.

In line with the Treaty on the Functioning of the European Union, we urge stakeholders across the European vaccination landscape to champion a future where health protection is paramount. By harnessing the full potential of technology in vaccine distribution, planning and evaluation, we can secure the well-being of Europe, fortify communities, and safeguard our socio-economy. This commitment will contribute to a resilient Europe that flourishes within an ethical framework that prioritises innovation, health, and prosperity for all its citizens.
